# Dr. Holmes on Specialism

**Published:** 1891-04

**Authors:** 


### SELECTIONS.

### Dr. Holmes on Specialism.

Oliver Wendell Holmes puts in the mouth of a young doctor the following words: "I am very glad," he said, "that we have a number of practitioners among us who confine themselves to the care of single organs and their functions. I want to be able to consult an oculist who has done nothing but attend to eyes long enough to know all that is known about their diseases and their treatment; skilful enough to be trusted with the manipulation of that delicate and most precious organ. I want an aurist who knows all about the ear, and what can be done for its disorders. The maladies of the larynx are very ticklish things to handle, and nobody should be trusted to go behind the epiglottis who has not the *tactus eruditus.* And so of other particular classes of complaints. A great city must have a limited number of experts, each a final authority to be appealed to in cases where the family physician finds himself in doubt. There are operations which no surgeon should be willing to underake unless he has paid a particular, if not an exclusive attention to the cases demanding such operations. All this I willingly grant, but it must not be supposed that we can return to the methods of the old Egyptians, who, if my memory serves me correctly, had a special physician for every part of the body; in short, falling into certain errors, and incurring certain liabilities. The specialist is much like other people engaged in a lucrative business. He is apt to magnify his calling, and to make much of any symptom which will bring a patient within range of his battery of remedies. I found a case in one of our medical journals a couple of years ago which illustrates what I mean. Dr. ——, of Philadelphia, had a female patient with a crooked nose—deviated septum, if our young scholars like that better. She was suffering from what the doctors call reflex headache. She had been to an oculist, and found that the trouble was her eyes. She went from him to a gynecologist who considered her headache was owing to causes for which his specialty had remedies How many more specialists would have appropriated her if she had gone the rounds of them all, I dare not guess; but you remember the siege in which each artisan proposed means of defense, which he himself was ready to furnish. Then a shoemaker said, 'Hang your walls with new boots!' Human nature is the same with the medical specialist as it was with ancient cordwainers, and it is possible, too, that a hungry practitioner may be warped by his interest in fastening on a patient, who, as he persuades himself, comes under his medical jurisdiction. The specialist has but one fang with which to seize and hold his prey; but that fang is a fearfully long and sharp canine. Being confined to a narrow field of observation and practice he is apt to give much of his time to curions study, which may be *magnifique*, but it is not exactly *la guerre* against the patient's malady. He divides and subdivides, and gets many varieties of diseases, in most respects similar. These he quips with new names, and thus we have those terrific nomenclatures which are enough to frighten the medical student, to say nothing of the sufferers staggering under this long catalogue of local infirmities. The 'old fogie' doctor who knows the family tendencies of his patient, who understands his constitution, will often treat him better than the famous specialist who sees him for the first time, and has to guess at many things. The old doctor knows from his previous experience with the same patient and the family to which he belongs. It is a great luxury to practice as a specialist in almost any class of diseases. The special practitioner has his own hours, hardly needs a night-bell, can have his residence out of the town in which he exercises his calling, in short lives like a gentleman, while the hard-working general practitioner submits to a servitude more exacting than that of the man who is employed in his stable or kitchen. That is the kind of life I have made my mind up to."

For Bites of Mosquetoes and Fleas, also other eruptions attended with intense itching, use menthol in alcohol, one part to ten. Useful also in headache.

